# Cell sex affects extracellular matrix protein expression and proliferation of smooth muscle progenitor cells derived from human pluripotent stem cells

**DOI:** 10.1186/s13287-017-0606-2

**Published:** 2017-07-04

**Authors:** Yanhui Li, Yan Wen, Morgaine Green, Elise K. Cabral, Prachi Wani, Fan Zhang, Yi Wei, Thomas M. Baer, Bertha Chen

**Affiliations:** 1 Department of Obstetrics/Gynecology, Stanford University School of Medicine, 300 Pasteur Drive HH-333, Stanford, CA 94305 USA; 2 Department of Obstetrics/Gynecology, Union Hospital, Tongji Medical College, Huazhong University of Science and Technology, Wuhan, People’s Republic of China; 3 Stanford Photonics Research Center, Department of Applied Physics, Stanford University, Stanford, CA USA

**Keywords:** Sex differences, Smooth muscle progenitor cell, Pluripotent stem cell, Estrogen, Extracellular matrix, Cell proliferation, Cell differentiation

## Abstract

**Background:**

Smooth muscle progenitor cells (pSMCs) differentiated from human pluripotent stem cells (hPSCs) hold great promise for treating diseases or degenerative conditions involving smooth muscle pathologies. However, the therapeutic potential of pSMCs derived from men and women may be very different. Cell sex can exert a profound impact on the differentiation process of stem cells into somatic cells. In spite of advances in translation of stem cell technologies, the role of cell sex and the effect of sex hormones on the differentiation towards mesenchymal lineage pSMCs remain largely unexplored.

**Methods:**

Using a standard differentiation protocol, two human embryonic stem cell lines (one male line and one female line) and three induced pluripotent stem cell lines (one male line and two female lines) were differentiated into pSMCs. We examined differences in the differentiation of male and female hPSCs into pSMCs, and investigated the effect of 17β-estradiol (E2) on the extracellular matrix (ECM) metabolisms and cell proliferation rates of the pSMCs. Statistical analyses were performed by using Student’s *t* test or two-way ANOVA, *p* < 0.05.

**Results:**

Male and female hPSCs had similar differentiation efficiencies and generated morphologically comparable pSMCs under a standard differentiation protocol, but the derived pSMCs showed sex differences in expression of ECM proteins, such as MMP-2 and TIMP-1, and cell proliferation rates. E2 treatment induced the expression of myogenic gene markers and suppressed ECM degradation activities through reduction of MMP activity and increased expression of TIMP-1 in female pSMCs, but not in male pSMCs.

**Conclusions:**

hPSC-derived pSMCs from different sexes show differential expression of ECM proteins and proliferation rates. Estrogen appears to promote maturation and ECM protein expression in female pSMCs, but not in male pSMCs. These data suggest that intrinsic cell-sex differences may influence progenitor cell biology.

**Electronic supplementary material:**

The online version of this article (doi:10.1186/s13287-017-0606-2) contains supplementary material, which is available to authorized users.

## Background

Smooth muscle cells (SMCs) are vital in maintaining the structural and functional integrity of hollow organs. When SMCs are injured, rather than regenerating they are partially replaced by fibroblasts and scar tissue [1]. Over time, these injuries can result in compromised function and in a wide range of diseases involving the urological [2], cardiovascular [3], or gastrointestinal systems [4]. Therefore, SMCs are an attractive target for treatment in regenerative medicine. During embryonic development, endothelial cells in the nascent hollow organs recruit smooth muscle progenitor cells (pSMCs) to be invested in tubular walls [5]. Because of their unexhausted differentiation and proliferation potential, pSMCs are thought to have better therapeutic efficacy compared with mature SMCs [6, 7]. Mature SMCs isolated from donor tissues are insufficient in number for transplantation, have limited proliferative potential, and exhibit reduced protein synthesis during in-vitro culture [8]. Unfortunately, the existence of pSMCs in adult organs remains controversial and the number of resident pSMCs in reported studies is too low to be isolated and purified for regenerative therapy [9, 10].

With their unlimited self-renewal potential and the high differentiation capacity to various types of cells, human pluripotent stem cells (hPSCs) are now highlighted as promising cell sources for tackling diseases with smooth muscle pathologies [11]. Standard differentiation protocols allow for production of large numbers of pSMCs. We have successfully differentiated human embryonic stem cells (hESCs) and induced pluripotent stem cells (iPSCs) into functional pSMCs and SMCs [12, 13]. Not only can these hPSC-derived pSMCs and SMCs serve as cell sources for translational research, they can also be used to investigate SMC function and development. The derived pSMCs show ability to restore function to a damaged urethral sphincter in a rodent model [13]. Several pilot studies also suggest that pSMCs derived from embryonic stem cells (ESCs) may restore blood flow in ischemic tissues [6, 14, 15] and regenerate the smooth muscle layer in damaged lower urinary tract [16].

The therapeutic potential of pSMCs derived from different cell sources may be very different. For example, pSMCs derived from adult mesenchymal stem cells (MSCs) [17] suffer from limited proliferation potential and culture senescence during in-vitro differentiation, leading to difficulty in obtaining sufficient number of SMCs for clinical applications [18]. Reprogramming methods also have a profound impact on the SMC-committed differentiation capability of iPSCs [19]. Another important factor affecting stem cell differentiation and function may be cell sex [20], which is often ignored in many studies.

Sex differences are well documented in the prevalence, morbidity, and mortality of several human diseases with SMC dysfunctions, such as pulmonary arterial hypertension [21], abdominal aortic aneurysm [22], atherosclerosis, and pelvic floor disorders [23]. Sex differences have also been shown in cell therapy using adult stem cells or progenitor cells [24, 25] and in the differentiation of hPSCs into neurons [20]. These raise the question of whether sex differences exist in hPSC-derived pSMCs. It is reported that sex hormones regulate proliferation, survival, and function of terminally differentiated SMCs [26]. However, very little is known about the function of sex hormones on pSMCs. The availability of a homogeneous population of hPSC-derived pSMCs provides an opportunity to investigate the sex differences in SMC development, differentiation, and function. Identification of sex differences in hPSC-derived pSMCs provides the necessary basis for a better understanding of the physiological and pathological differences in smooth muscle tissues, and for optimization of gender-specific treatments in regenerative medicine with SMCs.

Therefore, in this study, using a homogeneous population of hPSC-derived pSMCs differentiated by a chemically defined protocol, we sought to explore differences in stem cell differentiation between male and female hPSCs and the effect of estrogen on hPSC-derived pSMCs.

## Methods

### Cell culture, differentiation, and estrogen stimulation

This study was approved by the Institutional Review Board of the Stanford University School of Medicine and the Stanford University Stem Cell Research Oversight Committee (reference number 350). All experimental methods were carried out in accordance with the approved guidelines. Two male hPSC lines (H1 ESCs (WA01; WiCell Research Institute) and HuF1-iPSCs) and three female hPSC lines (H9 ESCs (WA09; WiCell Research Institute), HuF3-iPSCs and HuF5-iPSCs) were used in this study. All iPSC lines were kind gifts from Dr Renee Reijo Pera’s laboratory and were reprogrammed by inducing expression of transcription factors *POF5F1*, *KLF4*, *SOX2*, and *c-MYC* using retrovirus vectors in healthy adult dermal fibroblasts [27]. Written informed consent was obtained from each subject. Specimens were handled and carried out in accordance with the approved guidelines. All iPSC lines are fully characterized. H1/H9 ESCs and iPSCs were maintained on SC-qualified Matrigel-coated (catalog no. 354277; BD Biosciences, San Diego, CA, USA) dishes in mTeSR1 (catalog no. 85851; StemCell Technologies, Vancouver, BC, Canada). Cells were routinely passaged using Accutase (catalog no. AT104100; Innovative Cell Technologies, Inc.) and replated as single cells at a dilution of 1:10–1:15. For pSMC differentiation, hPSCs were dissociated into single cells using Accutase and plated on Matrigel-coated dishes at a density of 10,000 cells/cm^2^ in mTeSR with 10 μM ROCK inhibitor Y-27632 (catalog no. C9127-2 s; Cellagen Technology, San Diego, CA, USA). After 48–72 h, the medium was replaced with a chemically defined medium, consisting of RPMI 1640 with 1 mM Glutamax, 1% nonessential amino acids (catalog no. 61870; Invitrogen, Carlsbad, CA, USA), 0.1 mM β-mercaptoethanol, 1% penicillin and streptomycin (catalog no. 15140-122; Invitrogen), 1% ITS (catalog no. I3146; Sigma-Aldrich, St. Louis, MO, USA) supplemented with 50 ng/ml Activin A, 50 ng/ml human bone morphogenetic protein 4 (BMP4) (catalog nos AF-120-14E and 120-05ET; PeproTech, Rocky Hill, NJ, USA) and 5 μM CHIR99021 (catalog no. S2924; Selleckchem, Houston, TX, USA) for 2 days, and then 50 ng/ml basic fibroblast growth factor (bFGF) and 40 ng/ml vascular endothelial growth factor (VEGF) (catalog nos 100-18B and 100-20; PeproTech) for 7 days. Nine days after differentiation, cells were dissociated with Accutase, labeled with FITC Mouse Anti-Human CD31 and PerCP-Cy™5.5 Mouse Anti-Human CD34 (catalog nos BDB555445 and BDB347203; BD Biosciences, San Jose, CA, USA) and then sorted through fluorescence activating cell sorter (FACS). CD31 and CD34 double-positive cells (named passage 0) were sorted and replated on collagen IV-coated six well plates in smooth muscle growth medium (catalog no. M-231-500; Invitrogen), supplemented with 10 ng/ml PDGF-BB (cat. no. 315-18-10UG; PeproTech). The medium was exchanged every day for 5 days.

For gene and protein expression assays, cells were subsequently passaged and replated on collagen IV-coated dishes at a density of 1 × 10^4^ cells/cm^2^ and treated with different concentrations of 17β-estradiol (E2; 0, 0.1, 1, and 10 nM) (catalog no. E8875; Sigma-Aldrich) for 14 days, at which time the derived pSMCs were at passage 1 at the beginning of stimulation and at passage 3 on day 14. For terminal SMC differentiation, the pSMCs at passage 4 were cultured in smooth muscle differentiation medium (catalog no. S0085; Invitrogen) for 5 days.

### Immunofluorescence staining

Differentiated cells were dissociated with 0.05% Trypsin–EDTA (catalog no. 25300062; Invitrogen) and replated on collagen IV-coated eight-well Lab-Tek chamber slides (catalog no. 154534; Nunc, Rochester, NY, USA) at a density of 2.5 × 10^5^ cells/cm^2^. After incubation for 24 h, cells were rinsed with PBS and fixed with 4% paraformaldehyde in PBS for 10 min at room temperature. The cells were then incubated for 5 min in 0.5% Triton X-100 and 1% bovine serum albumin (catalog no. NIST927E; Sigma-Aldrich) in 0.1% Triton X-100/PBS for permeabilization and blocking, respectively. The cells were then incubated with primary antibodies overnight at 4 °C, followed by appropriate secondary antibodies in a moisture chamber. 4,6-Diamidino-2-phenylindole (DAPI) was used as a nuclear counterstain. Images were obtained using a fluorescence microscope (DP71; Olympus, Tokyo, Japan). Primary antibodies against the following molecules were used: α-smooth muscle actin (1:100, rabbit polyclonal antibody, catalog no. ab15734; Abcam, Cambridge, MA, USA), SM22 alpha (1:50, goat polyclonal antibody, catalog no. ab10135; Abcam), desmin (1:40, mouse monoclonal antibody, catalog no. D1033; Sigma), calponin (1:100, rabbit monoclonal antibody, catalog no. ab46794; Abcam), estrogen receptor (ER)-α (1:15, mouse monoclonal antibody, catalog no. sc-8005; Santa Cruz, CA, USA) and ER-β (1:100, rabbit polyclonal antibody, catalog no. ab5786; Abcam). Secondary antibodies were goat anti-rabbit IgG-Alexa 488 (1:500, catalog no. A-11008; Invitrogen), donkey anti-goat IgG-FITC (1:300, catalog no. sc-2024; Santa Cruz), and goat anti-mouse IgG-Alexa Fluor 488 (1:300, catalog no. A32723; Invitrogen). Negative control experiments were carried out by replacing primary antibodies with 1% bovine serum albumin in 0.1% Triton X-100/PBS.

### Cell contraction studies

Cell contraction studies were conducted to verify SMC function. Cell contractions were induced by treating the cells with 100 μM carbachol (catalog no. Y0000113; Sigma-Aldrich). Videos were recorded every 1 min for 10 min on a Nikon Biostation IM (Nikon, Tokyo, Japan). ImageJ software was used to measure the cell surface area before and 10 min after carbachol treatment. For each cell, at least three measurements were performed and the mean of percent change in surface area was recorded. The mean of the percent change in surface area of cells from each group was used for comparisons between groups.

### RNA extraction and quantitative reverse transcription-polymerase chain reaction

Total RNA was extracted with the RNA-STAT-60 reagent (catalog no. Cs-110; Tel-Test, Inc., Friendswood, TX, USA). RNA yield was determined using a Nanodrop 2000 spectrophotometer (Thermo Scientific). Total RNA (1 μg) was reverse transcribed into cDNA using the M-MLV reverse transcriptase system (catalog no. 28025013; Thermo Scientific). PCR primers are presented in Table 1. GAPDH was used as an endogenous reference. Real-time quantitative reverse transcription-polymerase chain reaction (qRT-PCR) was carried out on the Mx3005P Multiplex Quantificative PCR System with MxPro QPCR software (Stratagene, La Jolla, CA, USA). Brilliant SYBR Green QPCR Master Mix (catalog no. 600828; Stratagene) was used to perform PCR. GAPDH was used as an endogenous reference against which the different template values were normalized. All PCR reactions were performed in duplicate. The cycle of threshold (Ct) method was used for quantification. Data were analyzed by MxPro QPCR software.

**Table 1:** Primers used for real-time quantitative reverse transcription-polymerase chain reaction

Gene	Strand	5′–3′ sequence	Accession number
*GAPDH*	Sense	CTCAACGACCACTTTGTCAAGCTCA	NC_000012.12
	Anti-sense	GGTCTTACTCCTTGGAGGCCATGTG	
*Hu-SMA-alpha*	Sense	CCAGTGTGGAGCAGCCCAGC	NC_000001.11
	Anti-sense	TCACCCCCTGATGTCTGGGACG	
*Hu-Smoothelin*	Sense	TTGGACAAGATGCTGGATCA	NC_000022.11
	Anti-sense	CGCTGGTCTCTCTTCCTTTG	
*SM22-alpha*	Sense	GCTTGGAGCCATCAGGGTA	NC_000011.10
	Anti-sense	GGAGTGGATCATAGTGCAGTGT	
*Collagen I-alpha1*	Sense	TGTCTTATGGCTATGATGAG	NC_000017.11
	Anti-sense	ATCCAAACCACTGAAACC	
*Collagen III-alpha1*	Sense	GGCTCCTGGTGAGCGAGGAC	NC_000002.12
	Anti-sense	CCCATTTGCACCAGGTTCTCC	
*Hu-Elasin*	Sense	GTCGCAGGTGTCCCTAGTGT	NC_000007.14
	Anti-sense	GGTCCCCACTCCGTACTTG	
*TIMP-1*	Sense	GGGCTTCACCAAGACCTACA	NC_000023.11
	Anti-sense	TGCAGGGGATGGATAAACAG	
*MMP-1*	Sense	AGTGACTGGGAAACCAGATGCTGA	NC_000011.10
	Anti-sense	TCTGCTTGACCCTCAGAGACC	
*MMP-2*	Sense	CGAATCCATGATGGAGAGGC	NC_000016.10
	Anti-sense	TCCGTCCTTACCGTCAAAGG	

GenBank accession numbers indicate transcript variants with homologous sequences to primers

### Gelatin zymography

After treatment with 17β-estradiol for 13 days in SMGS growth medium, pSMCs were washed twice with PBS and then cultured in serum-free medium 231 supplemented with 0.2% w/v lactalbumin enzymatic hydrolysate (catalog no. 68458-87-7; Sigma) and 17β-estradiol for 24 h. The cell culture supernatant was collected and further concentrated (20×) by ultrafiltration using centrifugal filter units with 10-kDa cutoff (catalog no. VS0403; Sartorius Stedim SUS Inc., CA, USA). Gelatinolytic activities of matrix metalloproteinases (MMPs) in cell culture supernatants were assessed by gelatin zymography. In brief, total protein concentrations were determined using the Bradford method (Bio-Rad, Hercules, CA, USA). Samples were mixed with nonreducing sample buffer before being electrophoresed in 8% (wt/vol) polyacrylamide gels containing 0.1% gelatin in the presence of SDS. After electrophoresis, the gels were washed twice with 2.5% Triton X-100 and were subsequently incubated overnight at 37 °C in the substrate buffer (containing 50 mM Tris–HCl, pH 8, 5 mM CaCl2, 0.02% Azide). After staining with Coomassie blue, enzyme activity appeared as clear bands against the blue-stained background. The activities of MMPs were identified by molecular weight. The area of lysis for each band detected was analyzed using Bio-Rad Quality One Software (Bio-Rad). The results are expressed as densitometric units (intensity/mm^2^).

### Western blot assay

After stimulation with 17β-estradiol at the concentrations indicated for 14 days, pSMCs were washed twice with cold PBS and homogenized on ice with a RIPA buffer (50 mM Tris, 150 mM NaCl, 1% NP40, 0.5% deoxycholate, 0.1% SDS, 4 mM EDTA, and 2 mM PMSF, pH 7.4) supplemented with proteinase inhibitor (Roche Diagnostics GmbH, Basel, Switzerland), and then rotated at 4 °C for 2 days to solubilize the protein more efficiently. The cell debris was removed by centrifugation at 14,000 rpm for 30 min. Total protein concentrations were determined using the Bradford method (Bio-Rad). The samples were not reduced for collagen analysis and were reduced for other protein analysis with a sodium dodecyl sulfate (SDS) sample buffer containing 5% of 2-mercaptoethanol and boiled for 7 min. The proteins (15 μg/lane) were subjected to 10% polyacrylamide gels (SDS-PAGE). The gels were blotted onto nitrocellulose membranes (Bio-Rad) in an electrophoretic transfer cell (Bio-Rad). Blots were blocked with 5% nonfat milk at 4 °C overnight, and then probed with mouse anti-ERα antibody (1:150, catalog no. sc-8005; Santa Cruz), rabbit anti-ERβ antibody (1:1000, cat. no. ab5786; Abcam), rabbit anti-collagen III antibody (1:2500, catalog no. ab7778; Abcam), mouse anti-TIMP-1 antibody (1:1000; Calbiochem, La Jolla, CA, USA), and mouse anti-TIMP-2 antibody (1:200; Calbiochem) at room temperature for 1 h. After washing three times with phosphate-buffered saline with 0.1% Tween-20, pH 7.4 (PBS-T), the membrane was then incubated with sheep anti-mouse IgG conjugated to HRP (1:5000, catalog no. RPN4201; GE Healthcare, Pittsburgh, PA, USA) or donkey anti-rabbit lgG conjugated to HRP (1:5000, cat. no. NA934V; GE Healthcare) for 1 h at room temperature, followed by three washes in PBS-T. Blots were developed by chemiluminescence. The blots were reprobed with goat anti-GAPDH polyclonal antibody (1/5000, catalog no. ab9483; Abcam) and then 1/5000 dilution of mouse anti-goat IgG conjugated to HRP (catalog no. 31400; Invitrogen). The band density was determined by ImageJ Software (National Institutes of Health, Bethesda, MD, USA).

### Cell proliferation/death analysis by time-lapse microscopy

Cell mitosis and cell death events in the presence or absence of E2 were determined by time-lapse imaging. This technique allowed us to monitor cell mitosis and death over a period of time without exposure to harmful light or radiation. hPSC-pSMCs at passage 3 were replated on collagen IV-coated 24-well plates at a density of 2 × 10^4^ cells/cm^2^ and treated with different concentrations of 17β-estradiol (0, 0.1, 1.0, and 10 nM). Time-lapse imaging was conducted the following day on an inverted modified wide-field Olympus IX-70/71 microscope with dark-field optics. This microscope is equipped with a heated stage and a CO_2_ chamber to ensure identical conditions to that of a standard incubator [28]. The time-lapse images were acquired every 5 min for 48 h. Acquired image data were analyzed by two experienced individuals (blinded to study groups) using ImageJ software as described by Mackay et al. [29]. Briefly, after opening the video in ImageJ, “plugins/analysis/cell counter” is selected in the menu bar. The video is advanced frame by frame, during which the operator identifies cells undergoing mitosis or cell death. The mitotic entry frame is defined as the first frame with a nucleus showing early prophase characteristics, while the dying entry frame is defined as the cells displaying characteristic morphology, such as cytoplasmic swelling and detachment. These events are tagged, followed in the subsequent frames, and then recorded by the software. Only those cells completing mitosis are recorded. For a new cell death to be recorded, it has to be in a different location than those noted in previous frames. For each video, at least three counts were performed by different operators and the mean was taken. There was close agreement between data from the two independent operators.

### Statistical analysis

Values represent mean ± SD from three independent experiments. Statistical significance was analyzed using Student’s *t* test or one-way and two-way ANOVA, when appropriate, with the Tukey post-hoc test for multiple comparisons. All data were analyzed using the software SPSS21.0 (IBM Corp., Armonk, NY, USA). A difference was considered statistically significant when *p* < 0.05.

## Results

### Differentiation of hPSCs into pSMCs

**Fig. 1:**
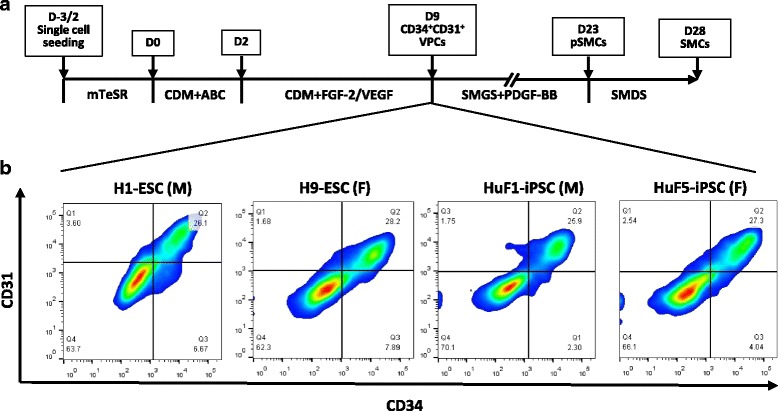
Differentiation efficiency of male and female hPSCs into smooth muscle progenitor cells (*pSMCs*). **a** Schematic of the protocol for differentiation of hPSCs to pSMCs and terminal differentiated SMCs. **b** Percentage of CD34^+^/CD31^+^ cells in the male and female hPSC derivatives. All hPSCs showed similar differentiation efficiency via a standardized differentiation protocol. *D* day, *CDM* chemically defined media, *ABC* Activin A, bone morphogenetic protein 4, and CHIR99021, *FGF* fibroblast growth factor, *VEGF* vascular endothelial growth factor, *SMGS* smooth muscle growth medium, *SMDS* smooth muscle differentiation medium, *iPSC* induced pluripotent stem cell, *ESC* embryonic stem cell

To examine the influence of cell sex on the differentiation capability of hPSCs, the hPSCs were induced toward the SMC fate. During in-vitro differentiation, we did not observe any differences between the male (*n* = 2) and the female (*n* = 3) lines, assessed by the percentage of the intermediate CD34^+^/CD31^+^ vascular progenitor cells (VPCs) and the cell morphological evolution during differentiation (Fig. 1b). These observations suggest that cell sex does not affect the differentiation capability of hPSCs into VPCs.

### Characterization of hPSC-derived pSMCs

**Fig. 2:**
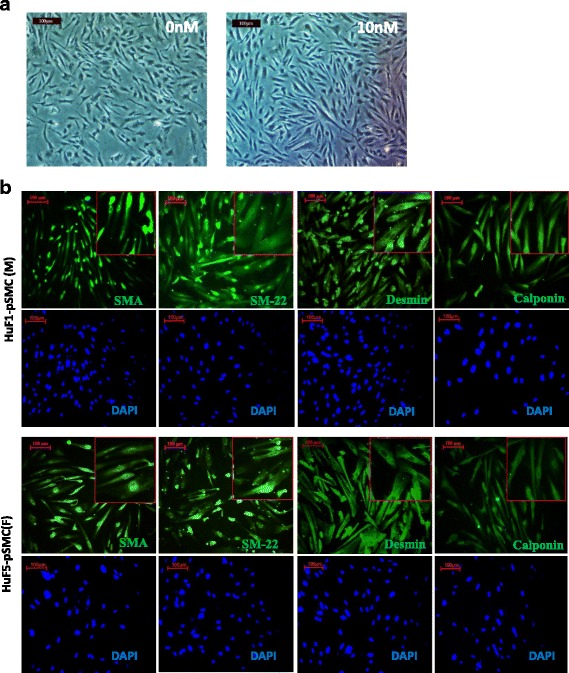
Characterization of smooth muscle progenitor cells (*pSMCs*) derived from male and female hPSCs. **a** Derived pSMCs exhibited the characteristic “spindle” appearance of SMC phenotype after culture in SMGS medium for 14 days. Cell morphology was unaffected by E2 treatment (0 and 10 nM) for 14 days. *Scale bar*, 100 μm. **b** Characterization of pSMCs derived from hPSCs by immunofluorescence staining. More than 95% of derived pSMCs expressed calponin and desmin, >90% SM-22, and >85% SMA when superimposed with DAPI. *Blue*, DAPI-stained nuclei. Representative image of three independent experiments. *Scale bar*, 100 μm. *SMA* smooth muscle actin, *DAPI* 4,6-diamidino-2-phenylindole, *F* female, *M* male (Color figure online)

All derived cells exhibited the characteristic spindle-shape appearance of SMCs after being cultured in SMGS medium on collagen IV-coated dishes for 14 days (Fig. 2a). The expression of SMC proteins was confirmed with immunofluorescence staining, which revealed robust expression of α-smooth muscle actin (α-SMA), SM22-α, desmin, and calponin (Fig. 2b). Greater than 85% of cells stained positively for SMC proteins. This pattern of staining was seen in all five pSMC lines.

To verify the ability of hPSC-pSMCs to differentiate into functional SMCs, male and female iPSC-pSMCs were further differentiated into terminal SMCs with smooth muscle differentiation media for 5 days, and then stimulated with carbachol for cell contraction studies. More than 70% of cells contracted with carbachol and these exhibited a change of 24.2 ± 18.5% in cell surface area (*n* = 15) (Additional file 1: Movie S1). These results are comparable to our previous studies using other hESC/iPSC lines and human aorta SMCs [12]. In addition, there were no significant differences in contractility between male and female pSMCs.


Additional file 1: Movie S1. Cell contraction assay. Terminally differentiated SMCs presented in this movie were derived from HuF1-iPSCs. (AVI 3664 kb)


### Expression of estrogen receptor in hPSC-derived pSMCs

**Fig. 3:**
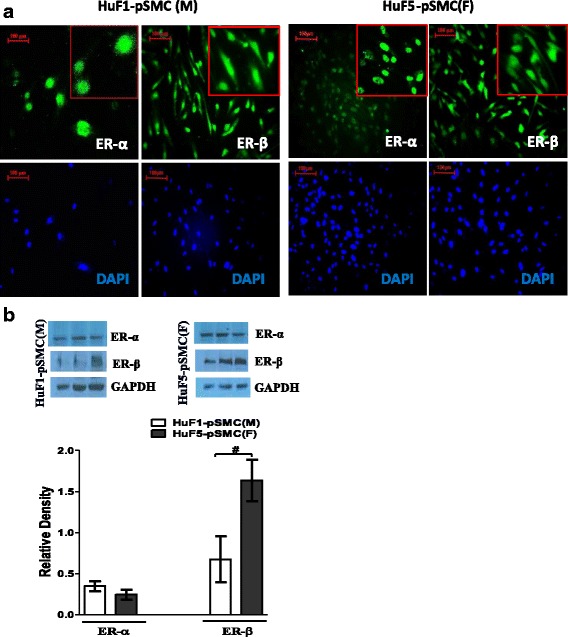
Expression of estrogen receptors in male and female smooth muscle progenitor cells (*pSMCs*). **a** Immunofluorescence staining showed that iPSC-derived pSMCs stained positively for ER-α and ER-β. **b** Western blot analysis for ER-α and ER-β protein in male HuF1-pSMCs and female HuF5-pSMCs. HuF1-pSMCs and HuF5-pSMCs expressed equivalent amounts of ER-α, while the expression of ER-β in female iPSC-pSMCs showed greater than twofold increase relative to that in male iPSC-pSMCs. Representative blots from three separate experiments performed in triplicate. ^**#**^ *p* < 0.05 by Student’s *t* test. Data shown represent the mean ± SD from three independent experiments, each performed in triplicate. *ER* estrogen receptor, *DAPI* 4,6-diamidino-2-phenylindole, *F* female, *M* male

Sex hormones exert their effect through interactions with their nuclear receptors. Estrogen receptors (ERs) are expressed in the developing embryoid bodies and various adult stem/progenitor cells, and may be involved in the sex dimorphism of stem/progenitor cells. Previous studies have documented the presence of ER-α and ER-β in hESCs [30]. We confirmed that our male and female iPSC-derived pSMCs also expressed both ER-α and ER-β by immunofluorescence staining and western blot assay (Fig. 3a, b). Of note, although male and female pSMCs expressed equivalent amounts of ERα, the expression of ER-β showed greater than twofold increase in female pSMCs relative to that in male pSMCs (*p* < 0.05) (Fig. 3b). Full-length blots are presented in Additional file 2: Figure S1.

### Estrogen induces the expression of myogenic markers in pSMCs

**Fig. 4:**
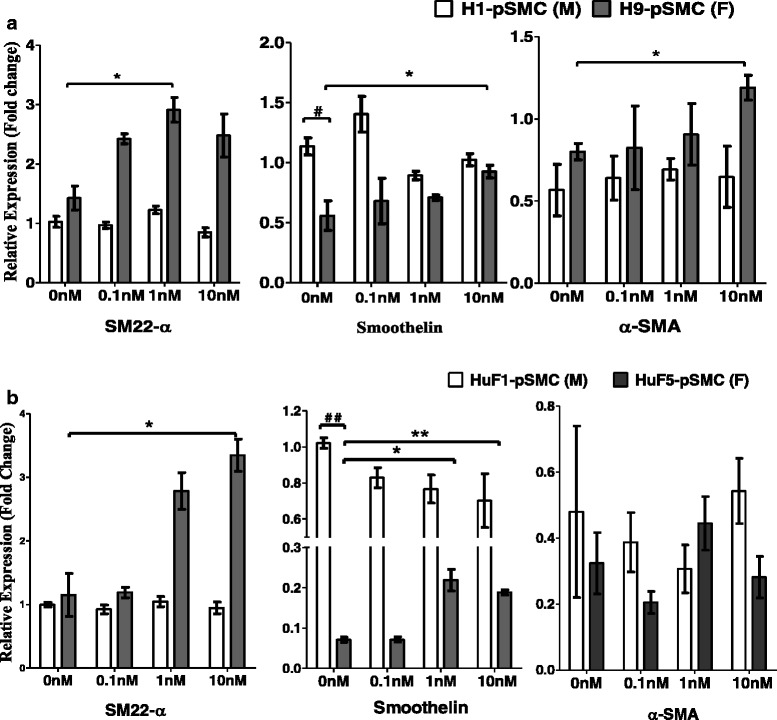
E2 stimulation induces the expression of myogenic marker in female smooth muscle progenitor cells (*pSMCs*) (**a**, **b**). Expression levels of SMC-specific genes were analyzed by quantitative real-time RT-PCR in male and female pSMCs. E2 promoted the gene expression of myogenic markers, including SM22, smoothelin, and/or α-SMA in female H9-pSMCs and HuF5-pSMCs (*p < 0.05 vs untreated cells), but not in male H1-pSMCs and HuF1-pSMCs. Student’s *t* test indicates significantly lower smoothelin expression at baseline (no estrogen stimulation) in female compared to male cell lines (^**#**^ *p* < 0.05, ^**##**^ *p* < 0.01). Estrogen stimulation data analyzed by one-way and two-way ANOVA followed by Tukey post-hoc test. Data shown represent the mean ± SD from three independent experiments, each performed in duplicate. *F* female, *M* male, *SMA* smooth muscle actin

At baseline (no estrogen stimulation), there was robust gene expression of SMC-specific markers (α-SMA, smoothelin, and SM22-α) in both male and female hPSC-derived pSMCs. No significant sex differences were detected with respect to gene expression of SM22-α and α-SMA (*p* > 0.05), but gene expression of smoothelin was significantly higher in male pSMCs compared to female pSMCs for both hESC and iPSC pairs (*p* < 0.05, Fig. 4a, b).

Given the estrogen-loaded environment in early human development in utero, we treated the pSMCs with physiologic concentrations of estrogen (E2) for 14 days to investigate the effect of E2 on the differentiation of the intermediate cell type from VPCs toward pSMCs. The mRNA levels of smoothelin and SM22-α increased in a dose-dependent fashion in response to E2 stimulation in female pSMCs (*p* < 0.05), but no effect was seen in the male pSMCs, suggesting that E2 exposure preferentially induces the myogenic gene markers in female pSMCs (Fig. 4a, b).

Because E2 stimulation during VPC differentiation to pSMCs enhanced the gene expression of SM-22α and smoothelin in female pSMCs, we also examined whether this effect is also present during terminal differentiation from pSMCs to SMCs. The gene expressions of SM-22α and smoothelin were not affected by E2 in the terminally differentiation step from pSMCs to SMCs (*p* > 0.05). Additional file 3: Figure S2 shows this in more detail.

### Extracellular matrix metabolism in hPSC-derived pSMCs

**Fig. 5:**
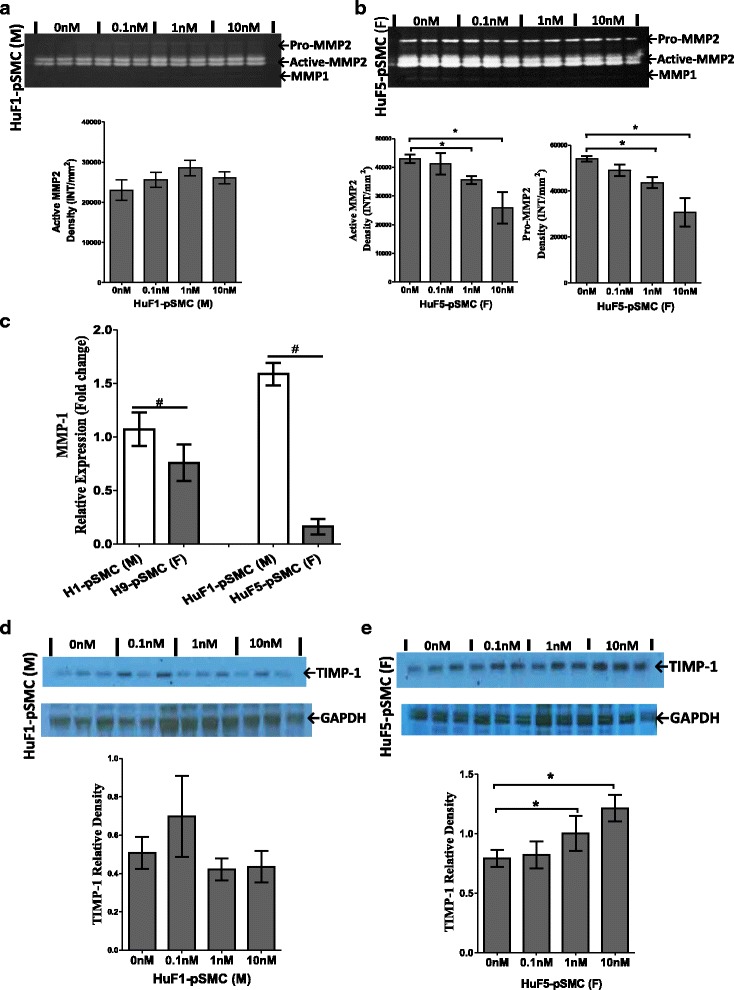
Sex differences in the activities and expression of ECM proteases of hPSC-derived smooth muscle progenitor cells (*pSMCs*) (**a**, **b**). Zymographic evaluation of MMP activities in concentrated condition media of male pSMCs (**a**) and female pSMCs (**b**) cocultured with different concentrations of 17β-estradiol. Zymograms are representative of three separate experiments. MMP-2 appeared as an active isoform (64/62 kDa) and a latent isoform (pro-MMP-2, 72 kDa), while MPP-1 appeared as an active isoform (43 kDa) in the gels. Graphs show the densitometric data of active-MMP-2 and/or pro-MMP-2 activities. **c** qRT-PCR analysis for the gene expression of MMP-1 in the male and female pSMCs at baseline (no E2 stimulation). **d**, **e** Western blot analysis of TIMP-1 protein in the concentrated supernatant from male and female pSMCs. Blots show changes in TIMP-1 protein in the concentrated condition media from male HuF1-pSMCs and female HuF5-pSMCs cocultured with or without E2. Graphs show the densitometric analysis of TIMP-1 protein expression in each group respectively. Results were normalized with internal control GAPDH. Data shown represent the mean ± SD from three independent experiments, each performed in triplicate (**a**, **b**, **d**, **e**) or duplicate (**c**). *p < 0.05, vs untreated pSMCs. ^**#**^ *p* < 0.05, male pSMCs vs female pSMCs. *F* female, *M* male, *MMP* matrix metalloproteinase, *TIMP* tissue inhibitor of metalloproteinase

In addition to their contractile function, SMCs are known to synthesize extracellular proteins which modulate extracellular matrix (ECM) in the surrounding tissue environment [31]. To assess differences in cell function, we examined the effect of cell sex and E2 stimulation on the activities of MMPs in concentrated cell culture supernatant via nonreducing gelatin zymography. At baseline, supernatant from iPSC-derived pSMCs showed marked MMP-2 gelatinolytic activity and absent MMP-1 activity (Fig. 5a, b). hESC-derived pSMCs showed a similar pattern in MMP-1 and MMP-2 activity. Additional file 4: Figure S3B, S3C shows this in more detail. Because MMP-1 activity was not detectable by zymography, we examined MMP-1 gene expression. Gene expression of MMP-1 in male pSMCs derived from hESC and iPSCs was significantly higher than that in the corresponding female pSMCs (*p* < 0.05) (Fig. 5c). Full-length blots are presented in Additional file 2: Figure S1.

With E2 stimulation, MMP-2 activity in the supernatants decreased significantly in H1/9-hESC-pSMCs and female iPSC-pSMCs (*p* < 0.05), but not in the male HuF1-iPSC-pSMCs (Fig. 5a, b; Additional file 4: Figure S3A, S3B, S3C). No change in MMP-1 activity was seen with E2 stimulation of H1/9-ESC-pSMCs (Additional file 4: Figure S3B, S3C). MMP-1 activity in the supernatants of HuF1/3/5-iPSC-pSMCs was too low to be quantified (Fig. 5a, b; Additional file 4: Figure S3A).

Because activity of MMPs is tightly regulated by their physiological inhibitors, the tissue inhibitor of metalloproteinases (TIMPs), we also examined their presence in the supernatants. Western blot assay demonstrated that E2 stimulation significantly increased the secretion of TIMP-1 protein in female pSMCs (*p* < 0.05), but had no significant effect on male pSMCs (Fig. 5d, e; Additional file 4: Figure: S3D, S3E). Taken together, these results suggest that E2 stimulation of female hPSC-derived pSMCs alters ECM metabolism. The decrease in MMP-2 activity in combination with increased expression of the protease inhibitor, TIMP-1, indicates that female pSMCs respond to E2 stimulation by inhibiting ECM degradation. In contrast, male pSMCs revealed decreased/no change in MMP-2 activity and no change in TIMP-1 expression. This pattern suggests that estrogen may not have the same effect on MMP-2/TIMP-1 modulation in the male pSMCs.

### Collagen I/III expression in hPSC-derived pSMCs

**Fig. 6:**
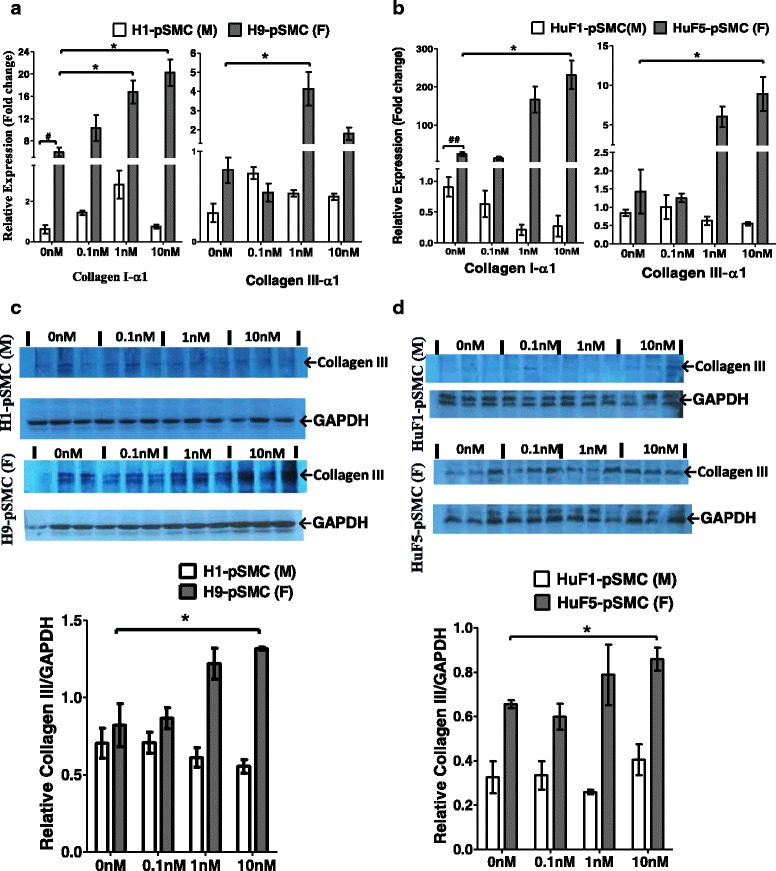
Effect of cell sex on the expression of ECM proteins. **a**, **b** Expression levels of collagen IαI and collagen IIIαI genes in two pairs of pSMCs were examined by qRT-PCR. At baseline (no E2), female pSMCs had a significantly higher expression of collagen IαI gene, compared to the corresponding male pSMCs. E2 treatment induced collagen IαI and collagen IIIαI gene expression in female pSMCs in a dose-dependent manner, but not in male pSMCs. **c**, **d** Western blot analysis of collagen III protein in male and female pSMCs. E2 treatment induced collage IIIαI expression in female pSMCs, but not in male pSMCs. *p < 0.05, vs untreated cells; data analyzed by one-way and two-way ANOVA followed by Tukey post-hoc test. ^**#**^ *p* < 0.05, male pSMCs vs female pSMCs by Student’s *t* test. Data shown represent the mean ± SD from three independent experiments, each performed in duplicate (**a**, **b**) or triplicate (**c**, **d**). *pSMC* smooth muscle progenitor cell, *F* female, *M* male, *MMP* matrix metalloproteinase, *TIMP* tissue inhibitor of metalloproteinase

Consistent with previous observations that SMCs synthesize ECM components (including elastin, collagen I/III) in tubular organs [31, 32], pSMCs derived from hPSCs showed high gene expression levels of collagen Iα1 and collagen IIIα1, and a relatively low expression of elastin gene. At baseline, female pSMCs derived from both hESCs and iPSCs had significantly higher gene expression of collagen Iα1 compared to their male counterparts (*p* < 0.05, Fig. 6a, b). Full-length blots are presented in Additional file 2: Figure S1.

E2 stimulation significantly increased the mRNA levels of collagen Iα1 and collagen IIIα1 in female pSMCs (*p* < 0.01), but not in male pSMCs (Fig. 6a, b). Likewise, E2 stimulation significantly promoted the expression of collagen III protein in female H9-ESC-pSMCs and female iPSC-pSMCs (*p* < 0.05), but had no effect on their male counterparts (Fig. 6c, d). Elastin mRNA expression was unaffected by E2 stimulation in either male or female hPSC-derived pSMCs. Additional file 5: Figure S4 shows this in more detail.

### Cell mitosis/death in hPSC-derived pSMCs

**Fig. 7:**
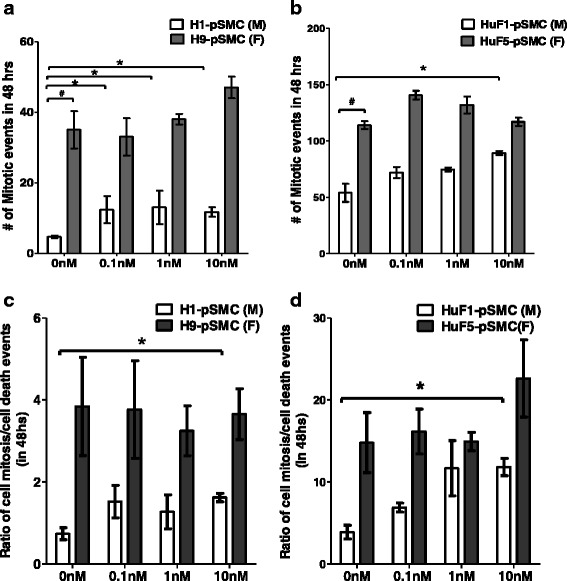
Effect of cell sex on cell proliferation of hPSC-derived smooth muscle progenitor cells (*pSMCs*). **a**, **b** Cell mitosis of male and female pSMCs was monitored with time-lapse dark-field microscopy for 48 h. Graphs show the total (cumulative) number of mitotic events of the male and female pSMCs cocultured with or without E2. **c**, **d** Cell mitosis/cell death ratio for hESC and iPSC male/female pairs. ^**#**^ *p* < 0.05 by Student’s *t* test, male pSMCs vs female pSMCs. *p < 0.05 vs untreated cells; data analyzed by two-way ANOVA followed by Tukey post-hoc test. Data shown represent the mean ± SD from three independent experiments, each performed in triplicate. *F* female, *M* male

Previous studies have reported that the mitotic rate may be different between male and female hPSCs [33, 34]. To investigate the effect of cell sex on cell proliferation during the intermediate stage of differentiation (from VPCs to pSMCs), we compared cell mitosis in two pairs of hPSC-derived pSMCs for 48 h using time-lapse dark-field microscopy. This technique allows exact quantification of cell proliferation and death over a long period of time without exposing the cells to harmful light or radiation. Without E2, female pSMCs derived from H9-ESCs and HuF5-iPSCs underwent more mitotic events during the 48-h observation period, compared to their male counterparts (*p* < 0.001, Fig. 7). Overall, the mitotic events were higher in iPSC groups compared to hESC groups, even when adjusted for cell death. E2 stimulation promoted cell proliferation in male pSMCs (*p* < 0.01), but not in female pSMCs. However, overall mitotic rates were higher in female compared to males (Fig. 7; Additional file 6: Movie S2 and Additional file 7: Movie S3). There were no significant differences in the cell death events with or without E2 for any of the cell types or sexes. Additional file 8: Figure S5, Additional file 6: Movie S2 and Additional file 7: Movie S3 show this in more detail.

## Discussion

Humans exhibit sex differences in various physiologic and pathologic conditions. Several diseases associated with SMC dysfunction show sex difference, including atherosclerosis [35], abdominal artery aneurysm [36], and stroke [37]*.* Previously, these sex differences were mainly attributed to the differential regulatory effects of the gonadal hormones. However, there is accumulating evidence that sex differences might not relate only to variations of circulating sex steroid levels, but also to the intrinsic differences of the target cell [38]. Functional SMCs have great therapeutic potential for restoring organ function and promoting tissue regeneration in the aforementioned diseases through transplantation or integration into tissue-engineered vessels [39, 40]. Little is known about whether sex differences exist in the differentiation of stem cells to the mesenchymal cell lineage and in the function of human pSMCs and SMCs resulting from this differentiation. In this study, using a standardized and clinical-grade differentiation protocol, we demonstrated that male and female hPSCs can be efficiently differentiated into pSMCs in vitro with nearly equal differentiation efficiencies. However, sex differences exist in the function and proliferation of hPSC-derived pSMCs in response to E2 stimulation.

It has been reported that stem cells derived from different tissue sources show marked variability in their ability to differentiate into pancreatic cells and cardiomyocytes [41]. To examine whether cell sex affects stem cell differentiation, we compared differentiation efficiency between male and female hPSCs using the same smooth muscle differentiation protocol. CD34^+^/CD31^+^ cells are known to give rise to vessels during embryonic development [42] and have been identified as VPCs which can generate SMCs via in-vitro directed differentiation [12, 43]. Therefore, the percentage of CD34^+^/CD31^+^ VPCs in the hPSC derivatives was used to compare SMC differentiation efficiency in male and female hPSCs. Our data show that both male and female hPSCs can be induced into VPCs with similar efficiencies, suggesting that cell sex has no effect on the pSMC-committed differentiation. This observation is in agreement with recent studies [44, 45] which, although not directly focusing on the role of cell sex in the stem cell differentiation, showed that SMCs can be efficiently generated from male and female hPSCs through similar differentiation protocols as ours.

Stem cell differentiation in sexually dimorphic mammalian organs (such as sexual glands) is regulated by sex hormone [38, 46]. An important question for translation of stem cell-based therapies is whether the differentiation of stem cells in organs that do not show sex-specific morphological differences, such as various hollow organs, is also influenced by circulating sex hormones. The role of E2 in the function, proliferation, and transdifferentiation of mature SMCs has been studied extensively. For example, E2 mediates airway SMC contraction by modulating intracellular Ca^2+^ [47] and an anti-proliferative effect of nitric oxide in female vascular SMCs [48]. Exogenous E2 induces the expression of α-actin in aortic SMCs and abolishes progressive growth of aortic aneurysms in female ovariectomized mice [49]. However, the effect of sex hormone on the differentiation of pSMCs has not been reported. The current study shows that exposure to E2 during the intermediate steps in the differentiation from hPSC to SMCs promotes gene expression of myogenic markers only in female hPSC-derived pSMCs. This is consistent with female animal data demonstrating that E2 treatment can promote the differentiation of female rat wall-resident CD34^+^ progenitor cells into vascular SMCs [50]. Our data also revealed that E2 treatment during the terminal differentiation step from pSMCs to SMCs did not affect gene expression of myogenic makers, suggesting that optimization of stem cell therapies for SMC with hormonal manipulation should be targeted at the intermediate stage of SMC differentiation. The lack of effect on SMC markers in the terminally differentiated SMCs may be due to saturation of these markers in the mature SMCs.

SMCs modulate ECM metabolism in tissues through synthesis of several types of ECM components (such as collagen I, collagen III, collagen IV, and fibronectin) and matrix remodeling enzymes such as matrix metalloproteinases (MMPs) and their inhibitors, the tissue inhibitors of MMPs (TIMPs) [31, 32]. MMP-1 and MMP-2 are capable of degrading multiple ECM components, including collagen and elastin [51]. Generally, all TIMPs are capable of inhibiting all known MMPs and ECM proteolysis. However, the efficacy of MMP inhibition varies with each TIMP. TIMP-1 is a strong inhibitor of many MMPs, except for some of the membrane type MMPs, while TIMP-2, TIMP-3, and TIMP-4 interact with pro-MMP-2. Elevated MMP-1 and MMP-2 have been strongly associated with ruptured atherosclerotic plaques and aortic aneurysms [52]. Thus, SMCs are important in maintaining the ECM homeostasis in hollow organs [31]. In this study, we identified that female pSMCs had a significantly lower expression of MMP-1 gene and a significantly higher expression of collagen I gene compared to male pSMCs. Furthermore, E2 stimulation decreases MMP-2 activity and increases TIMP-1 expression. Taken together, these findings suggest that female pSMCs may inhibit ECM proteolysis in an estrogen environment compared to male pSMCs. These differential cell responses to estrogen could contribute to the observed differences in cardiovascular morbidity between men and women, and can be optimized to enhance efficacy of cell replacement therapy and tissue-engineering applications. We note that MMP activity in the cell culture supernatants was analyzed by gelatin zymography. Although gelatin zymography is an excellent and simple tool for the identification of MMP activity, it is a semiquantitative technique [53] and does not reveal the integrated gelatinase activity in the cell culture media. Therefore, future in-vivo cell transplantation studies are needed to more accurately delineate the sex differences of pSMCs in ECM modulation.

Ability to generate a sufficient number of functional pSMCs is important for the translation of pSMC-based therapy. Using time-lapse microscopy, we found that female pSMCs proliferate more frequently than the corresponding male pSMCs. This observation is consistent with previous studies involving other stem/progenitor cell types [25, 54]. Another observation from our cell proliferation analysis is that the number of mitosis events in female HuF5-iPSC-pSMCs was significantly higher than that in female H9-ESC-pSMCs. Variability between different lines is likely due to intrinsic differences between individuals or to persistent epigenetic modifications in the iPSC lines. We do not believe the increase in proliferation rate in female iPSC-derived pSMCs is associated with persistent expression of pluripotency genes (*POF5F1*, *KLF4*, *SOX2*, and *c-MYC*) from the retroviral vector reprogramming technique, because our previous study showed that the FACS purification step for VPCs yields >99.9% purity with undetectable levels of these genes by PCR (in press). The undetectable but potential subpopulation of cells with residual pluripotency genes in the differentiated population would be too low to account for the significant increases in proliferation rates.

Our current study also showed that E2 affects proliferation of pSMCs in a sex-dependent manner. While female pSMCs exhibited higher overall mitotic events compared to male pSMCs, E2 stimulation only increased mitotic events in male pSMCs. It is interesting to note that while estrogen increased mitosis of male pSMCs, E2 treatment did not enhance the differentiation of male pSMCs from the VPC intermediate. Published data suggest that E2 can have different effects on CD34^+^ progenitor cells [50, 55]; it can accelerate the proliferation of undifferentiated CD34^+^ progenitor cells, while promoting further differentiation in differentiating progenitor cells. Thus, it is possible that there was a relatively higher fraction of undifferentiated VPCs that persisted in the male pSMC population compared to female pSMCs after FACS sorting. E2 stimulation of this small subpopulation increased proliferation, but did not increase differentiation from VPCs to pSMCs. Future studies involving detailed characterization of small subpopulations in the FACS sorted VPCs will help elucidate this possible effect.

Another possible confounder is that hPSCs can undergo genetic changes during in-vitro culture [56] which could affect proliferation. These changes have been documented in the H1/9 ESC lines [57]. Because these genetic aberrations are difficult to detect with karyotyping, a higher resolution technique is required to examine this source of error. Given the limited and exploratory nature of our studies, we opted to correlate the hESC data with data from iPSC lines, and found a similar pattern in both groups. Future studies are necessary to confirm the nature differential proliferation between sexes.

ER-α and ER-β are expressed in the early development embryoid bodies and modulate the differentiation of hESCs into various cell types [30]. There are robust data showing that physiologic levels of E2 can stimulate proliferation of hESCs, confirming an end-effect through interaction with ERs. We found that ER-α and ER-β were robustly expressed in the iPSC-derived pSMCs. Based on our limited sample size, it is not possible to determine whether there is differential expression of ER-α compared to ER-β. Data on the relative distribution of these receptors in human PSCs is scant due to limited available lines. Most studies document expression of these receptors, rather than relative expression. ER-β is also known to be involved in multiple aspects of SMC function and development, such as contraction, maturation, proliferation, and migration [58, 59]. It is possible that the effects observed in this study are the result of differential expression of ERs. Additional loss-of-function studies are needed to investigate the role of differential ER expression in modulating proliferation of pSMCs.

Finally, in-vivo animal studies are needed to confirm whether the observed cell-sex differences in pSMCs would affect the efficiency of cell therapy for conditions involving SMCs.

## Conclusion

pSMCs derived from male and female hPSCs show differential proliferation rates and expression of ECM proteins. Differences also exist in their response to estrogen during differentiation from VPCs to pSMCs. Estrogen appears to induce the expression of myogenic markers and ECM protein expression in female hPSC-derived pSMCs, but not in male pSMCs. In-vivo studies to examine whether these intrinsic cell-sex differences may influence clinical efficacy are necessary in the development of stem cell therapies.

## Additional files

Additional file 2: Figure S1. Showing full-length gel images. (PPTX 33934 kb)

Additional file 3: Figure S2. Showing the effect of E2 on the mRNA levels of SMC-specific markers in terminally differentiated SMCs. Terminally differentiated SMCs were derived from H9-ESCs. Expression levels of SMA-α, SM-22α, and smoothelin did not change significantly in the presence or absence of E2. Data analyzed by ANOVA followed by Tukey post-hoc test. Data shown represent the mean ± SD from three independent experiments, each performed in duplicate. (PPTX 86 kb)

Additional file 4: Figure S3. Showing sex differences in the expression of MMP-2 and TIMP-1 proteins in hPSC-derived pSMCs. (A), (B), (C) Gelatin zymography of MMP activities in concentrated condition media from HuF3-pSMCs, H1-pSMCs, and H9-pSMCs cocultured with different concentrations of 17β-estradiol. Graphs show the densitometric data of active-MMP-2 and/or pro-MMP-2 activities. (D), (E) Western blot analysis of TIMP-1 in the concentrated supernatants from male H1-pSMC and female H9-pSMCs. *p < 0.05, **p < 0.01, compared to untreated cells; data analyzed by two-way ANOVA followed by Tukey post-hoc test. Data shown represent the mean ± SD from three independent experiments, each performed in triplicate. (PPTX 415 kb)

Additional file 5: Figure S4. Showing E2 treatment on the expression of elastin gene in male and female hPSC-derived pSMCs. Expression level of elastin gene did not change significantly with E2 stimulation. Data analyzed by two-way ANOVA followed by Tukey post-hoc test. Data shown represent the mean ± SD from three independent experiments, each performed in duplicate. (PPTX 99 kb)

Additional file 6: Movie S2. showing cell mitosis analysis using time-lapse microscopy. pSMCs presented in this movie were derived from HuF1-iPSCs (male). HuF1-pSMCs were replated at a density of 2 × 10^4^ cells/cm^2^ and cocultured without 17β-estradiol. (AVI 11483 kb)

Additional file 7: Movie S3. showing cell mitosis analysis using time-lapse microscopy. pSMCs presented in this movie were derived from HuF5-iPSCs (female) and cultured in the SMGS media in the absence of 17β-estradiol. (AVI 10677 kb)

Additional file 8: Figure S5. Showing the effect of cell sex on cell death of hPSC-derived pSMCs. (A) hESC lines and (B) iPSC lines. Data analyzed by two-way ANOVA followed by Tukey post-hoc test. Data shown represent the mean ± SD from three independent experiments, each performed in triplicate. No statistical difference in cell death events was observed between female pSMCs and corresponding male pSMCs (*p* > 0.05). (PPTX 107 kb)

## Abbreviations

bFGF: Basic fibroblast growth factor
BMP4: Human bone morphogenetic protein 4
E2: 17β-estradiol
ECM: Extracellular matrix
ER: Estrogen receptor
FACS: Fluorescence activating cell sorter
hESC: Human embryonic stem cell
hPSC: Human pluripotent stem cell
iPSC: Induced pluripotent stem cell
MMP: Matrix metalloproteinase
PBST: Phosphate-buffered saline with 0.1% Tween-20
pSMC: Smooth muscle progenitor cell
qRT-PCR: Quantitative reverse transcription-polymerase chain reaction
SDS: Sodium dodecyl sulfate
SMC: Smooth muscle cell
TIMP: Tissue inhibitor of metalloproteinase
VEGF: Vascular endothelial growth factor
VPC: Vascular progenitor cell
SMA: Smooth muscle actin
