# Supplementary material for: Cell sex affects extracellular matrix protein expression and proliferation of smooth muscle progenitor cells derived from human pluripotent stem cells
Source: Stem Cell Res Ther. 2017 Jul 4;8:156. doi: 10.1186/s13287-017-0606-2 (PMC5496346; doi:10.1186/s13287-017-0606-2)
Supplement: Supplementary file 2 — Showing full-length gel images. (PPTX 33934 kb) [file 13287_2017_606_MOESM2_ESM.pptx]

## Slide 1
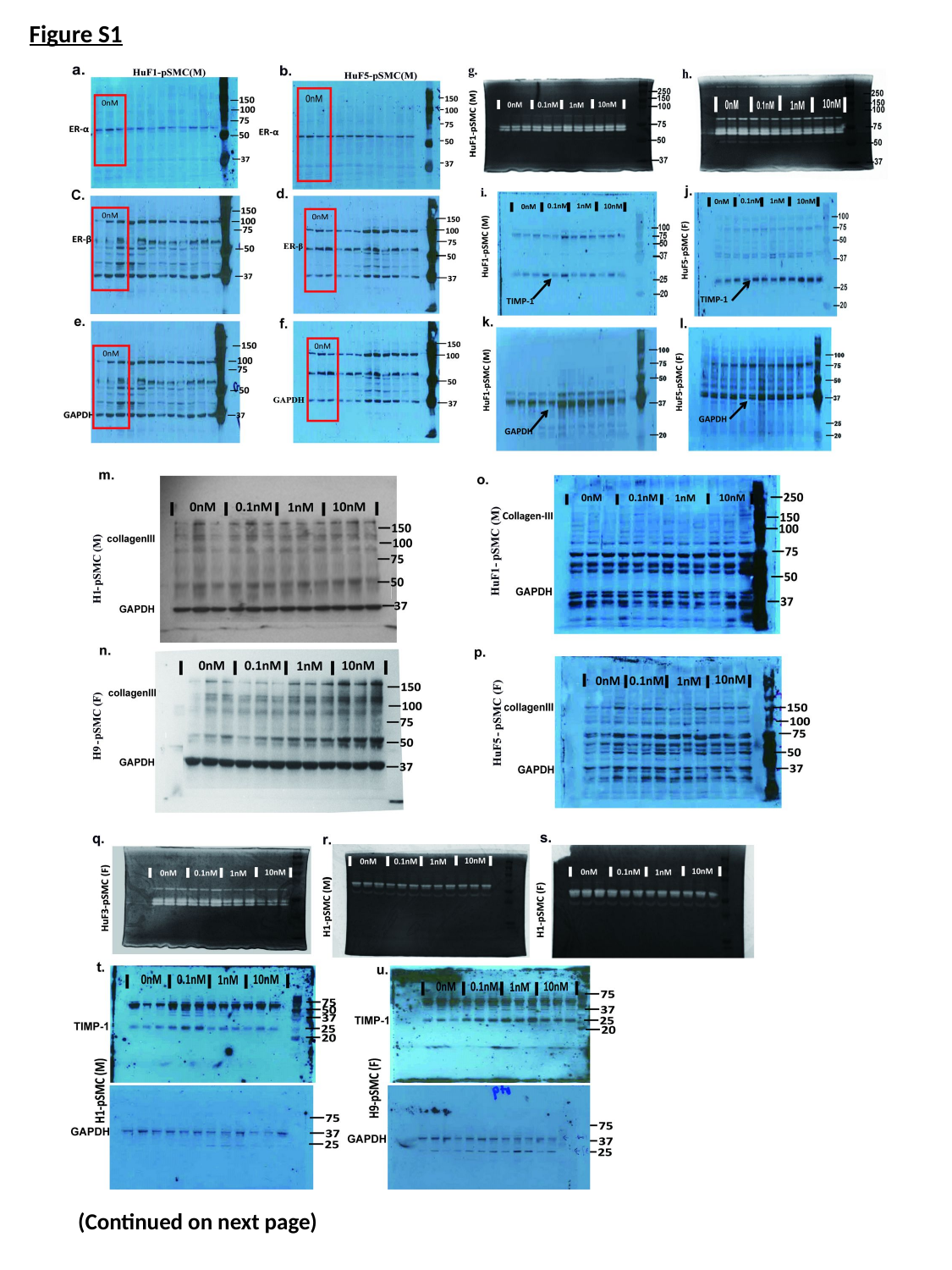

Figure S1
(Continued on next page)

## Slide 2
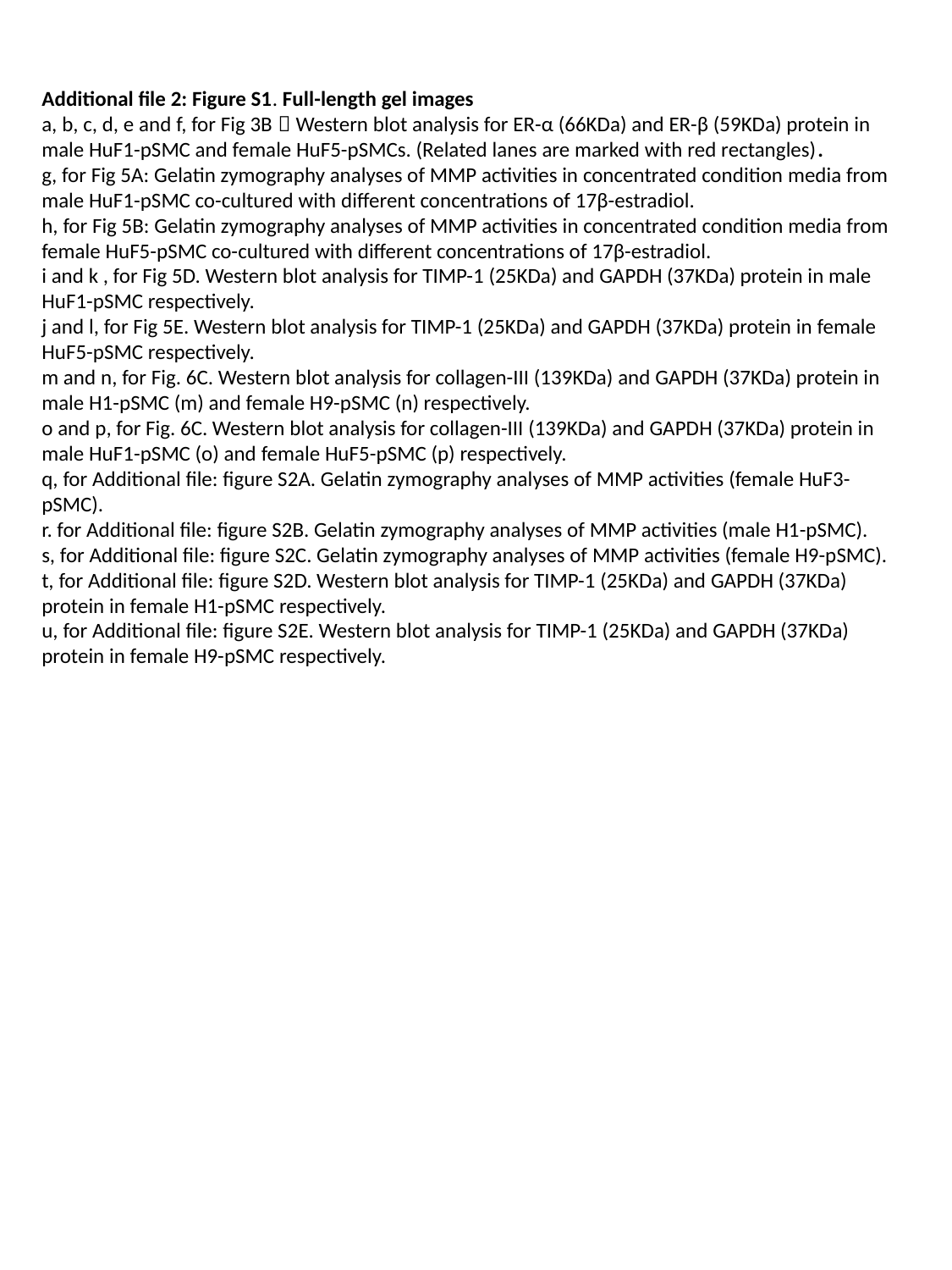

Additional file 2: Figure S1. Full-length gel images
a, b, c, d, e and f, for Fig 3B：Western blot analysis for ER-α (66KDa) and ER-β (59KDa) protein in male HuF1-pSMC and female HuF5-pSMCs. (Related lanes are marked with red rectangles).
g, for Fig 5A: Gelatin zymography analyses of MMP activities in concentrated condition media from male HuF1-pSMC co-cultured with different concentrations of 17β-estradiol.
h, for Fig 5B: Gelatin zymography analyses of MMP activities in concentrated condition media from female HuF5-pSMC co-cultured with different concentrations of 17β-estradiol.
i and k , for Fig 5D. Western blot analysis for TIMP-1 (25KDa) and GAPDH (37KDa) protein in male HuF1-pSMC respectively.
j and l, for Fig 5E. Western blot analysis for TIMP-1 (25KDa) and GAPDH (37KDa) protein in female HuF5-pSMC respectively.
m and n, for Fig. 6C. Western blot analysis for collagen-III (139KDa) and GAPDH (37KDa) protein in male H1-pSMC (m) and female H9-pSMC (n) respectively.
o and p, for Fig. 6C. Western blot analysis for collagen-III (139KDa) and GAPDH (37KDa) protein in male HuF1-pSMC (o) and female HuF5-pSMC (p) respectively.
q, for Additional file: figure S2A. Gelatin zymography analyses of MMP activities (female HuF3-pSMC).
r. for Additional file: figure S2B. Gelatin zymography analyses of MMP activities (male H1-pSMC).
s, for Additional file: figure S2C. Gelatin zymography analyses of MMP activities (female H9-pSMC).
t, for Additional file: figure S2D. Western blot analysis for TIMP-1 (25KDa) and GAPDH (37KDa) protein in female H1-pSMC respectively.
u, for Additional file: figure S2E. Western blot analysis for TIMP-1 (25KDa) and GAPDH (37KDa) protein in female H9-pSMC respectively.
