# Supplementary material for: Cell sex affects extracellular matrix protein expression and proliferation of smooth muscle progenitor cells derived from human pluripotent stem cells
Source: Stem Cell Res Ther. 2017 Jul 4;8:156. doi: 10.1186/s13287-017-0606-2 (PMC5496346; doi:10.1186/s13287-017-0606-2)
Supplement: Supplementary file 3 — Showing the effect of E2 on the mRNA levels of SMC-specific markers in terminally differentiated SMCs. Terminally differentiated SMCs were derived from H9-ESCs. Expression levels of SMA-α, SM-22α, and smoothelin did not change significantly in the presence or absence of E2. Data analyzed by ANOVA followed by Tukey post-hoc test. Data shown represent the mean ± SD from three independent experiments, each performed in duplicate. (PPTX 86 kb) [file 13287_2017_606_MOESM3_ESM.pptx]

## Slide 1
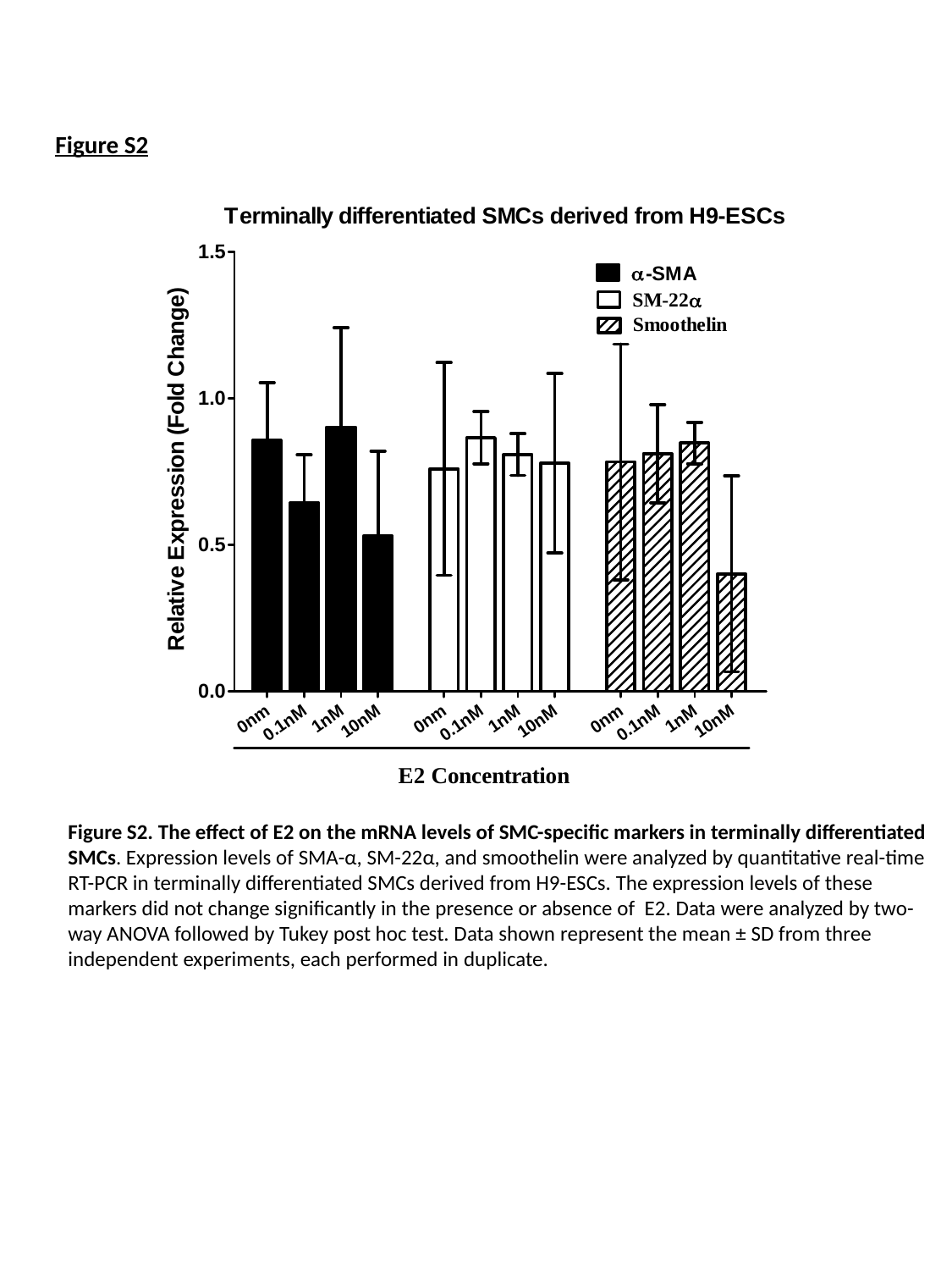

Figure S2
Figure S2. The effect of E2 on the mRNA levels of SMC-specific markers in terminally differentiated SMCs. Expression levels of SMA-α, SM-22α, and smoothelin were analyzed by quantitative real-time RT-PCR in terminally differentiated SMCs derived from H9-ESCs. The expression levels of these markers did not change significantly in the presence or absence of E2. Data were analyzed by two-way ANOVA followed by Tukey post hoc test. Data shown represent the mean ± SD from three independent experiments, each performed in duplicate.
