# Supplementary material for: Cell sex affects extracellular matrix protein expression and proliferation of smooth muscle progenitor cells derived from human pluripotent stem cells
Source: Stem Cell Res Ther. 2017 Jul 4;8:156. doi: 10.1186/s13287-017-0606-2 (PMC5496346; doi:10.1186/s13287-017-0606-2)
Supplement: Supplementary file 4 — Showing sex differences in the expression of MMP-2 and TIMP-1 proteins in hPSC-derived pSMCs. (A), (B), (C) Gelatin zymography of MMP activities in concentrated condition media from HuF3-pSMCs, H1-pSMCs, and H9-pSMCs cocultured with different concentrations of 17β-estradiol. Graphs show the densitometric data of active-MMP-2 and/or pro-MMP-2 activities. (D), (E) Western blot analysis of TIMP-1 in the concentrated supernatants from male H1-pSMC and female H9-pSMCs. *p < 0.05, **p < 0.01, compared to untreated cells; data analyzed by two-way ANOVA followed by Tukey post-hoc test. Data shown represent the mean ± SD from three independent experiments, each performed in triplicate. (PPTX 415 kb) [file 13287_2017_606_MOESM4_ESM.pptx]

## Slide 1
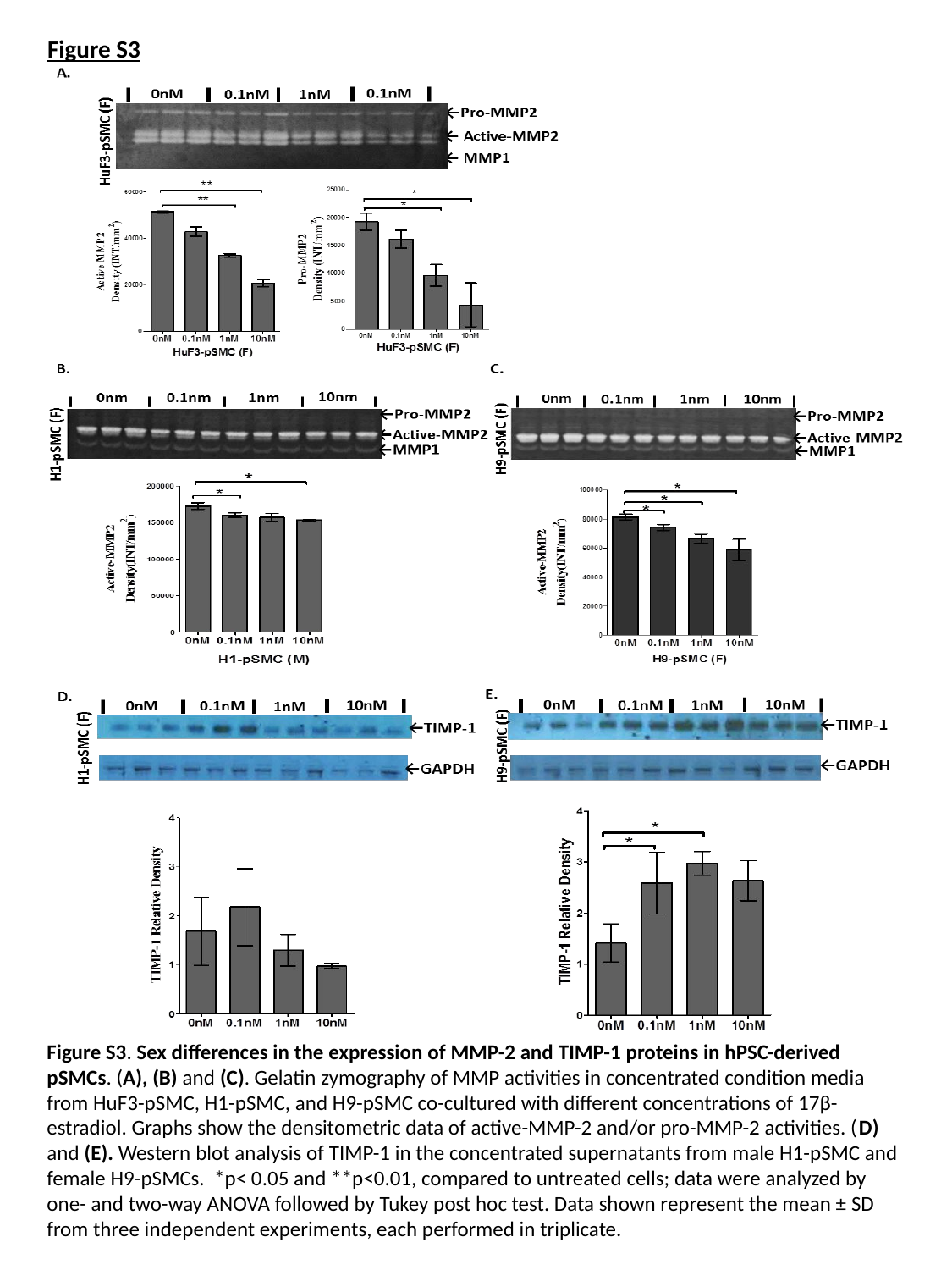

Figure S3
Figure S3. Sex differences in the expression of MMP-2 and TIMP-1 proteins in hPSC-derived pSMCs. (A), (B) and (C). Gelatin zymography of MMP activities in concentrated condition media from HuF3-pSMC, H1-pSMC, and H9-pSMC co-cultured with different concentrations of 17β-estradiol. Graphs show the densitometric data of active-MMP-2 and/or pro-MMP-2 activities. (D) and (E). Western blot analysis of TIMP-1 in the concentrated supernatants from male H1-pSMC and female H9-pSMCs. *p< 0.05 and **p<0.01, compared to untreated cells; data were analyzed by one- and two-way ANOVA followed by Tukey post hoc test. Data shown represent the mean ± SD from three independent experiments, each performed in triplicate.
