# Supplementary material for: Cell sex affects extracellular matrix protein expression and proliferation of smooth muscle progenitor cells derived from human pluripotent stem cells
Source: Stem Cell Res Ther. 2017 Jul 4;8:156. doi: 10.1186/s13287-017-0606-2 (PMC5496346; doi:10.1186/s13287-017-0606-2)
Supplement: Supplementary file 5 — Showing E2 treatment on the expression of elastin gene in male and female hPSC-derived pSMCs. Expression level of elastin gene did not change significantly with E2 stimulation. Data analyzed by two-way ANOVA followed by Tukey post-hoc test. Data shown represent the mean ± SD from three independent experiments, each performed in duplicate. (PPTX 99 kb) [file 13287_2017_606_MOESM5_ESM.pptx]

## Slide 1
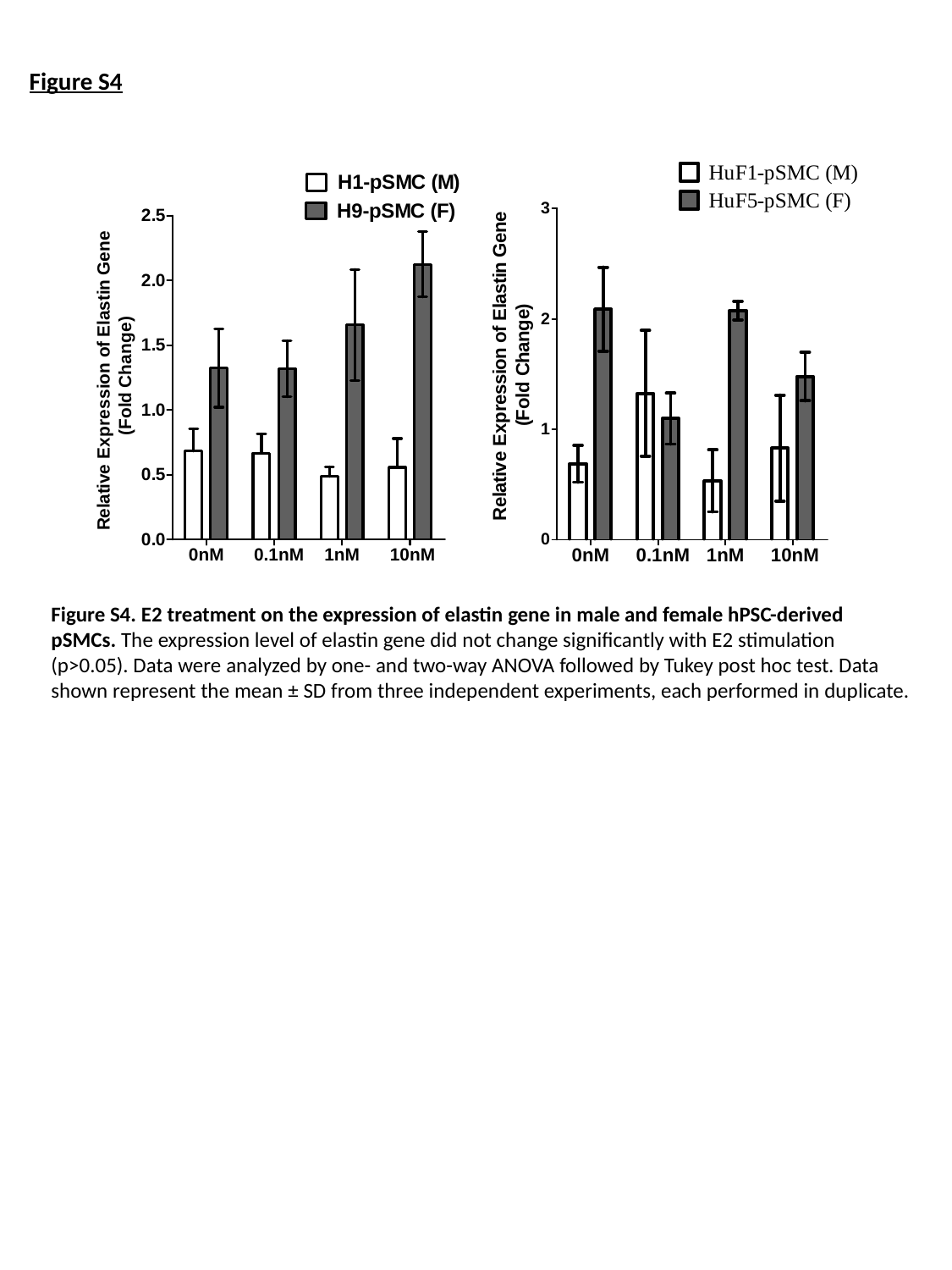

Figure S4
Figure S4. E2 treatment on the expression of elastin gene in male and female hPSC-derived pSMCs. The expression level of elastin gene did not change significantly with E2 stimulation (p>0.05). Data were analyzed by one- and two-way ANOVA followed by Tukey post hoc test. Data shown represent the mean ± SD from three independent experiments, each performed in duplicate.
