# Supplementary material for: Cell sex affects extracellular matrix protein expression and proliferation of smooth muscle progenitor cells derived from human pluripotent stem cells
Source: Stem Cell Res Ther. 2017 Jul 4;8:156. doi: 10.1186/s13287-017-0606-2 (PMC5496346; doi:10.1186/s13287-017-0606-2)
Supplement: Supplementary file 8 — Showing the effect of cell sex on cell death of hPSC-derived pSMCs. (A) hESC lines and (B) iPSC lines. Data analyzed by two-way ANOVA followed by Tukey post-hoc test. Data shown represent the mean ± SD from three independent experiments, each performed in triplicate. No statistical difference in cell death events was observed between female pSMCs and corresponding male pSMCs (p > 0.05). (PPTX 107 kb) [file 13287_2017_606_MOESM8_ESM.pptx]

## Slide 1
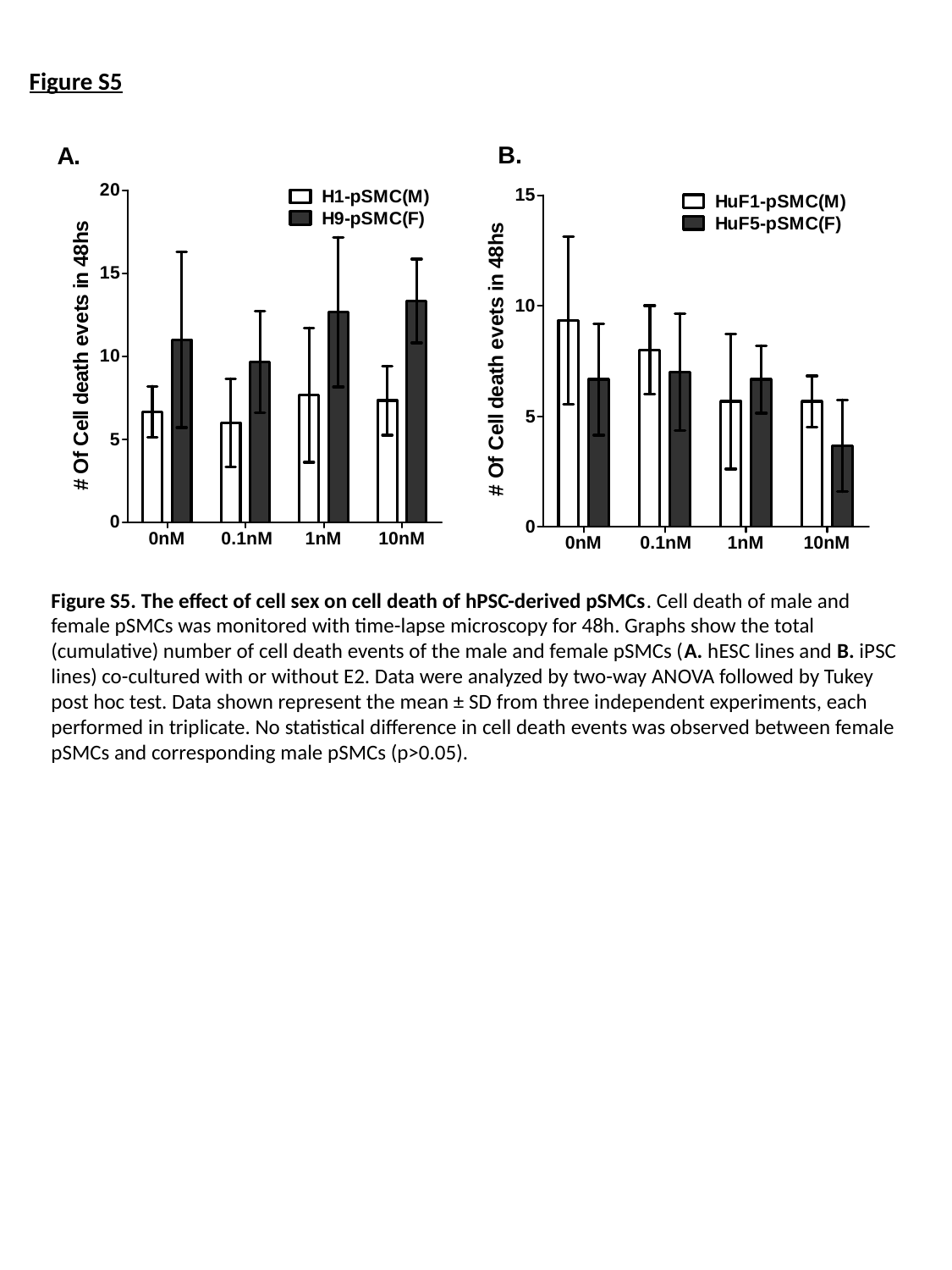

Figure S5
Figure S5. The effect of cell sex on cell death of hPSC-derived pSMCs. Cell death of male and female pSMCs was monitored with time-lapse microscopy for 48h. Graphs show the total (cumulative) number of cell death events of the male and female pSMCs (A. hESC lines and B. iPSC lines) co-cultured with or without E2. Data were analyzed by two-way ANOVA followed by Tukey post hoc test. Data shown represent the mean ± SD from three independent experiments, each performed in triplicate. No statistical difference in cell death events was observed between female pSMCs and corresponding male pSMCs (p>0.05).
